# Supplementary material for: Availability of alternative prey rather than intraguild interactions determines the local abundance of two understudied and threatened small carnivore species
Source: PLoS One. 2024 Nov 8;19(11):e0310021. doi: 10.1371/journal.pone.0310021 (PMC11548751; doi:10.1371/journal.pone.0310021)
Supplement: S1 Table — (DOCX) [file pone.0310021.s002.docx]

**S1 Table. Variance inflation factor (VIF) values for covariates used in the modeling framework at the three surveyed seasons.**

| **Covariates^a^** | **Dry season 2019** | **Rainy season 2019** | **Dry season 2020** |
| --- | --- | --- | --- |
| Competitor | 2.485 | 3.063 | 2.436 |
| Coyotes | 1.327 | 1.537 | 4.406 |
| Dogs | 2.053 | 1.973 | 2.568 |
| Ocelots | 1.712 | 1.620 | 2.966 |
| Burrows | 1.276 | 1.517 | 1.871 |
| Avasmam | 2.992 | 3.642 | 3.438 |
| Soilhum | 3.187 | 2.197 | 2.314 |
| Dishwater | 1.480 | 1.576 | 3.427 |
| Shrcover | 1.368 | 1.510 | 2.518 |
| Cancover | 2.826 | 1.334 | 2.994 |
| Effort | 1.287 | 1.963 | 2.881 |

^a^The key to covariate abbreviations is: competitor, presence of competitors; coyotes, presence of coyotes; dogs, presence of dogs; ocelots, presence of ocelots; burrows, potential burrows; avasmam, availability of small mammals; soilhum, soil humidity; dishwater, distance to the nearest water source; shrcover, shrub cover; cancover, canopy cover; effort, sampling effort.
