# Supplementary material for: Availability of alternative prey rather than intraguild interactions determines the local abundance of two understudied and threatened small carnivore species
Source: PLoS One. 2024 Nov 8;19(11):e0310021. doi: 10.1371/journal.pone.0310021 (PMC11548751; doi:10.1371/journal.pone.0310021)
Supplement: S2 Table — Selection of candidate Bayesian Generalized Linear Models explaining the effect of surveyed season and study zone on the abundances of skunk species (A) and the relationship in abundances between skunk species during the surveyed seasons in each study zone (B) using leave-one-out cross-validation for pairwise model comparisons. (DOCX) [file pone.0310021.s003.docx]

**S2 Table. Selection of candidate Bayesian Generalized Linear Models explaining the effect of surveyed season and study zone on the abundances of skunk species (A) and the relationship in abundances between skunk species during the surveyed seasons in each study zone (B)** **using leave-one-out cross-validation for pairwise model comparisons.**

|  | **Model** | **elpd** | **Δelpd** | **SE [Δelpd]** | **ω** |
| --- | --- | --- | --- | --- | --- |
| **A. Effect of surveyed seasons and study zones** | | | | | |
| **American hog-nosed skunk** | Poisson | -161.836 | 0.000 | 0.000 | 0.728 |
|  | Negative Binomial | -163.088 | -1.253 | 2.721 | 0.272 |
| **Pygmy spotted skunk** | Negative Binomial | -209.025 | 0.000 | 0.000 | 0.749 |
|  | Poisson | -215.738 | -6.713 | 5.578 | 0.251 |
| **B. Relationship in abundance between species** | | | | | |
| **Dry season 2019-DZ** | Poisson | -42.006 | 0.000 | 0.000 | 0.981 |
|  | Negative Binomial | -44.135 | -2.129 | 1.714 | 0.019 |
| **Rainy season 2019-DZ** | Negative Binomial | -52.978 | 0.000 | 0.000 | 0.757 |
|  | Poisson | -54.489 | -1.510 | 2.482 | 0.243 |
| **Dry season 2020-DZ** | Negative Binomial | -45.576 | 0.000 | 0.000 | 0.777 |
|  | Poisson | -47.581 | -2.005 | 2.834 | 0.223 |
| **Dry season 2019-PZ** | Negative Binomial | -20.549 | 0.000 | 0.000 | 1.000 |
|  | Poisson | -28.367 | -7.828 | 4.969 | 0.000 |
| **Rainy season 2019-PZ** | Poisson | -35.222 | 0.000 | 0.000 | 1.000 |
|  | Negative Binomial | -38.982 | -3.761 | 0.473 | 0.000 |

elpd = expected log pointwise predictive density; Δelpd = pairwise differences in elpd (relative to the top model); SE[Δelpd] = standard error of Δelpd; ω = model weight; DZ, Disturbed Zone; PZ, Protected Zone.
