# Supplementary material for: Availability of alternative prey rather than intraguild interactions determines the local abundance of two understudied and threatened small carnivore species
Source: PLoS One. 2024 Nov 8;19(11):e0310021. doi: 10.1371/journal.pone.0310021 (PMC11548751; doi:10.1371/journal.pone.0310021)
Supplement: S3 Table — (DOCX) [file pone.0310021.s004.docx]

**S3 Table. Parameter estimates of the best-ranked Bayesian Generalized Linear Models explaining the relationships in abundance between skunk species during the surveyed seasons in each study zone.**

|  | **Parameter** | **β** | **SD** | **2.5%** | **97.5%** | **MCSE** | **n_eff** | **Rhat** |
| --- | --- | --- | --- | --- | --- | --- | --- | --- |
| **Dry season 2019 - DZ** | Intercept | 0.665 | 0.361 | -0.060 | 1.373 | 0.007 | 2818 | 1 |
|  | Abund_hnskunk | -0.037 | 0.128 | -0.296 | 0.214 | 0.002 | 2929 | 1 |
|  | Mean_PPD | 1.809 | 0.375 | 1.154 | 2.578 | 0.006 | 3390 | 1 |
|  | Log-posterior | -43.478 | 1.003 | -46.267 | -42.493 | 0.027 | 1431 | 1 |
| **Rainy season 2019 - DZ** | Intercept | 0.553 | 0.258 | 0.041 | 1.046 | 0.005 | 3257 | 1 |
|  | Abund_hnskunk | 0.186 | 0.076 | 0.044 | 0.345 | 0.001 | 3329 | 1 |
|  | Reciprocal dispersion^a^ | 3.038 | 1.183 | 1.302 | 6.001 | 0.021 | 3172 | 1 |
|  | Mean_PPD | 3.001 | 0.810 | 1.731 | 4.846 | 0.014 | 3386 | 1 |
|  | Log-posterior | -56.844 | 1.219 | -59.891 | -55.438 | 0.029 | 1746 | 1 |
| **Dry season 2020 - DZ** | Intercept | 0.756 | 0.364 | 0.043 | 1.448 | 0.006 | 3636 | 1 |
|  | Abund_hnskunk | -0.084 | 0.193 | -0.464 | 0.306 | 0.003 | 3533 | 1 |
|  | Reciprocal dispersion | 1.997 | 0.951 | 0.749 | 4.415 | 0.017 | 3028 | 1 |
|  | Mean_PPD | 1.966 | 0.645 | 0.958 | 3.458 | 0.012 | 2700 | 1 |
|  | Log-posterior | -48.737 | 1.273 | -52.070 | -47.300 | 0.032 | 1565 | 1 |
| **Dry season 2019 - PZ** | Intercept | -1.005 | 0.996 | -2.628 | 1.348 | 0.022 | 1990 | 1 |
|  | Abund_hnskunk | 1.223 | 1.561 | -1.678 | 4.660 | 0.035 | 2000 | 1 |
|  | Reciprocal dispersion | 0.273 | 0.313 | 0.041 | 1.074 | 0.009 | 1345 | 1 |
|  | Mean_PPD | 2.818 | 34.340 | 0.048 | 8.668 | 0.738 | 2166 | 1 |
|  | Log-posterior | -23.028 | 1.452 | -26.711 | -21.338 | 0.044 | 1092 | 1 |
| **Rainy season 2019 - PZ** | Intercept | 0.668 | 0.159 | 0.349 | 0.964 | 0.003 | 2120 | 1 |
|  | Abund_hnskunk | 0.704 | 0.188 | 0.329 | 1.056 | 0.004 | 2127 | 1 |
|  | Mean_PPD | 2.366 | 0.462 | 1.500 | 3.318 | 0.008 | 3036 | 1 |
|  | Log-posterior | -36.786 | 0.972 | -39.405 | -35.805 | 0.022 | 1923 | 1 |

SD, Standard Deviation; MCSE, Monte Carlo standard error; n_eff, effective sample size; Rhat, diagnostic statistic (< 1.1); DZ, Disturbed Zone; PZ, Protected Zone; Abund_hnskunk, Abundance of hog-nosed skunks; Mean_PPD, sample average posterior predictive distribution of the outcome.

^a^ Smaller values of the parameter indicate greater dispersion [1]. 1. Gabry J, Goodrich B. Package ‘rstanarm’. Bayesian applied regression modeling via Stan. R package version 2.32.1. 2024. https://cran.r-project.org/web/packages/rstanarm/index.html
