# Supplementary material for: Availability of alternative prey rather than intraguild interactions determines the local abundance of two understudied and threatened small carnivore species
Source: PLoS One. 2024 Nov 8;19(11):e0310021. doi: 10.1371/journal.pone.0310021 (PMC11548751; doi:10.1371/journal.pone.0310021)
Supplement: S4 Table — (DOCX) [file pone.0310021.s005.docx]

|  | **American hog-nosed skunk** | | | | | **Pygmy spotted skunk** | | | | |
| --- | --- | --- | --- | --- | --- | --- | --- | --- | --- | --- |
|  | **Model**^a^ | **elpd** | **Δelpd** | **SE [Δelpd]** | **ω** | **Model**^a^ | **elpd** | **Δelpd** | **SE [Δelpd]** | **ω** |
| **Dry season 2019** | r_(.)_ | -43.833 | 0.000 | 0.000 | 1.000 | r_(effort + lunillu)_ | -152.012 | 0.000 | 0.000 | 0.528 |
|  | r_(effort)_ | -44.624 | -0.791 | 0.275 | 0.000 | r_(effort)_ | -152.119 | -0.107 | 1.120 | 0.074 |
|  | r_(lunillu)_ | -44.907 | -1.074 | 0.650 | 0.000 | r_(lunillu)_ | -152.158 | -0.145 | 2.620 | 0.135 |
|  | r_(effort + lunillu)_ | -45.830 | -1.997 | 0.803 | 0.000 | r_(.)_ | -152.581 | -0.569 | 2.996 | 0.263 |
| **Rainy season 2019** | r_(effort)_ | -79.999 | 0.000 | 0.000 | 0.633 | r_(lunillu)_ | -211.041 | 0.000 | 0.000 | 0.942 |
|  | r_(effort + lunillu)_ | -80.320 | -0.321 | 1.473 | 0.367 | r_(effort + lunillu)_ | -211.494 | -0.453 | 1.029 | 0.058 |
|  | r_(.)_ | -81.597 | -1.598 | 1.115 | 0.000 | r_(.)_ | -212.604 | -1.563 | 1.437 | 0.000 |
|  | r_(lunillu)_ | -82.116 | -2.117 | 1.228 | 0.000 | r_(effort)_ | -212.732 | -1.690 | 1.633 | 0.000 |
| **Dry season 2020** | r_(.)_ | -30.030 | 0.000 | 0.000 | 1.000 | r_(effort)_ | -79.492 | 0.000 | 0.000 | 0.939 |
|  | r_(lunillu)_ | -30.695 | -0.666 | 0.290 | 0.000 | r_(effort + lunillu)_ | -80.462 | -0.971 | 0.347 | 0.000 |
|  | r_(effort)_ | -30.945 | -0.916 | 0.393 | 0.000 | r_(.)_ | -80.760 | -1.269 | 1.772 | 0.061 |
|  | r_(effort + lunillu)_ | -31.558 | -1.528 | 0.539 | 0.000 | r_(lunillu)_ | -81.485 | -1.993 | 1.791 | 0.000 |

**S4 Table. Selection of candidate Royle-Nichols models explaining the detection probability (r) for skunk species during the surveyed seasons using leave-one-out cross-validation for pairwise model comparisons.**

elpd = expected log pointwise predictive density; Δelpd = pairwise differences in elpd (relative to the top model); SE[Δelpd] = standard error of Δelpd; ω = model weight.

^a^The key to covariate abbreviations is: effort, sampling effort; lunillu, lunar illumination.
