# Supplementary material for: Availability of alternative prey rather than intraguild interactions determines the local abundance of two understudied and threatened small carnivore species
Source: PLoS One. 2024 Nov 8;19(11):e0310021. doi: 10.1371/journal.pone.0310021 (PMC11548751; doi:10.1371/journal.pone.0310021)
Supplement: S5 Table — (DOCX) [file pone.0310021.s006.docx]

**S5 Table. Selection of candidate Royle-Nichols models explaining the abundance (λ) of skunk species from a priori hypotheses by three subsets of variables during the surveyed seasons using leave-one-out cross-validation for pairwise model comparisons.**

|  | **American hog-nosed skunk** | | | | | **Pygmy spotted skunk** | | | | |
| --- | --- | --- | --- | --- | --- | --- | --- | --- | --- | --- |
|  | **Model^a^** | **elpd** | **Δelpd** | **SE [Δelpd]** | **ω** | **Model^a^** | **elpd** | **Δelpd** | **SE [Δelpd]** | **ω** |
| **Dry season 2019** | *Interespecific interactions* | | | | |  |  |  |  |  |
|  | λ_(coyotes)_ | -44.533 | 0.000 | 0.000 | 0.555 | λ_(ocelots)_ | -151.631 | 0.000 | 0.000 | 0.534 |
|  | λ_(competitor)_ | -44.657 | -0.124 | 1.139 | 0.445 | λ_(competitor)_ | -151.894 | -0.264 | 3.205 | 0.465 |
|  | λ_(ocelots)_ | -44.869 | -0.336 | 0.714 | 0.000 | λ_(coyotes + ocelots)_ | -152.651 | -1.020 | 0.319 | 0.000 |
|  | λ_(dogs)_ | -44.956 | -0.424 | 0.793 | 0.000 | λ_(dogs)_ | -152.852 | -1.221 | 2.716 | 0.000 |
|  | λ_(coyotes + ocelots)_ | -45.215 | -0.682 | 0.617 | 0.000 | λ_(coyotes + ocelots + dogs)_ | -152.903 | -1.272 | 0.764 | 0.000 |
|  | λ_(coyotes + ocelots + dogs)_ | -46.380 | -1.848 | 0.983 | 0.000 | λ_(coyotes)_ | -153.460 | -1.830 | 2.341 | 0.000 |
|  | λ_(competitor + coyotes + ocelots + dogs)_ | -46.830 | -2.298 | 1.426 | 0.000 | λ_(competitor + coyotes + ocelots + dogs)_ | -153.785 | -2.154 | 2.773 | 0.000 |
|  | *Resource availability and habitat complexity* | | | | |  |  |  |  |  |
|  | λ_(diswater)_ | -41.220 | 0.000 | 0.000 | 1.000 | λ_(avasmam + diswater)_ | -143.540 | 0.000 | 0.000 | 0.680 |
|  | λ_(shrcover + cancover + burrows + avasmam + soilhum + diswater)_ | -43.366 | -2.146 | 1.804 | 0.000 | λ_(burrows + avasmam + soilhum + diswater)_ | -146.205 | -2.665 | 2.002 | 0.000 |
|  | λ_(burrows + avasmam + soilhum + diswater)_ | -43.375 | -2.155 | 1.032 | 0.000 | λ_(avasmam + soilhum)_ | -148.184 | -4.644 | 4.200 | 0.154 |
|  | λ_(shrcover + cancover)_ | -44.104 | -2.884 | 1.732 | 0.000 | λ_(shrcover + cancover + burrows + avasmam + soilhum + diswater)_ | -148.454 | -4.914 | 3.315 | 0.000 |
|  | λ_(shrcover + cancover + burrows)_ | -45.334 | -4.114 | 2.169 | 0.000 | λ_(diswater)_ | -149.997 | -6.457 | 5.447 | 0.166 |
|  | λ_(avasmam + soilhum)_ | -45.532 | -4.312 | 2.019 | 0.000 | λ_(shrcover + cancover + burrows)_ | -154.176 | -10.636 | 5.663 | 0.000 |
|  |  |  |  |  |  | λ_(shrcover + cancover)_ | -154.402 | -10.862 | 4.915 | 0.000 |
|  | *Combination* | | | | |  |  |  |  |  |
|  | λ_(diswater + coyotes)_ | -41.944 | 0.000 | 0.000 | 0.766 | λ_(avasmam + diswater + ocelots)_ | -143.865 | 0.000 | 0.000 | 0.644 |
|  | λ_(shrcover + cancover + avasmam + diswater + coyotes + ocelots)_ | -42.611 | -0.667 | 1.562 | 0.234 | λ_(burrows + avasmam + competitor)_ | -147.853 | -3.988 | 4.670 | 0.226 |
|  | λ_(shrcover + cancover + competitor)_ | -45.026 | -3.083 | 2.230 | 0.000 | λ_(avasmam + coyotes + ocelots)_ | -147.985 | -4.120 | 3.839 | 0.033 |
|  | λ_(avasmam + coyotes + ocelots)_ | -45.982 | -4.039 | 2.081 | 0.000 | λ_(shrcover + cancover + avasmam + diswater + coyotes + ocelots)_ | -148.560 | -4.695 | 3.001 | 0.000 |
|  | λ_(shrcover + cancover + coyotes + ocelots)_ | -46.068 | -4.124 | 2.031 | 0.000 | λ_(shrcover + cancover + avasmam + burrows + competitor)_ | -148.964 | -5.099 | 4.784 | 0.000 |
|  | λ_(shrcover + cancover + avasmam + burrows + competitor)_ | -46.700 | -4.756 | 2.150 | 0.000 | λ_(shrcover + cancover + competitor)_ | -153.658 | -9.793 | 5.692 | 0.096 |
|  | λ_(burrows + avasmam + competitor)_ | -47.234 | -5.290 | 2.586 | 0.000 | λ_(shrcover + cancover + coyotes + ocelots)_ | -154.774 | -10.909 | 4.666 | 0.000 |
| **Rainy season 2019** | *Interespecific interactions* | | | | |  |  |  |  |  |
|  | λ_(ocelots)_ | -80.569 | 0.000 | 0.000 | 0.924 | λ_(coyotes)_ | -211.216 | 0.000 | 0.000 | 0.611 |
|  | λ_(dogs)_ | -81.001 | -0.432 | 0.594 | 0.076 | λ_(ocelots)_ | -211.682 | -0.466 | 1.873 | 0.265 |
|  | λ_(coyotes)_ | -81.160 | -0.591 | 0.458 | 0.000 | λ_(dogs)_ | -211.955 | -0.739 | 1.921 | 0.001 |
|  | λ_(coyotes + ocelots)_ | -81.521 | -0.952 | 1.017 | 0.000 | λ_(coyotes + ocelots)_ | -212.068 | -0.852 | 1.226 | 0.000 |
|  | λ_(competitor)_ | -81.547 | -0.978 | 0.978 | 0.000 | λ_(competitor)_ | -212.172 | -0.956 | 2.139 | 0.122 |
|  | λ_(coyotes + ocelots + dogs)_ | -82.501 | -1.932 | 1.107 | 0.000 | λ_(coyotes + ocelots + dogs)_ | -212.279 | -1.063 | 1.384 | 0.000 |
|  | λ_(competitor + coyotes + ocelots + dogs)_ | -84.288 | -3.719 | 1.615 | 0.000 | λ_(competitor + coyotes + ocelots + dogs)_ | -213.446 | -2.231 | 1.707 | 0.000 |
|  | *Resource availability and habitat complexity* | | | | |  |  |  |  |  |
|  | λ_(cancover + avasmam + soilhum)_ | -73.593 | 0.000 | 0.000 | 1.000 | λ_(avasmam + diswater)_ | -193.749 | 0.000 | 0.000 | 0.926 |
|  | λ_(shrcover + cancover + burrows + avasmam + soilhum + diswater)_ | -75.545 | -1.952 | 1.235 | 0.000 | λ_(avasmam)_ | -194.454 | -0.704 | 1.279 | 0.074 |
|  | λ_(shrcover + cancover + burrows)_ | -77.248 | -3.655 | 2.357 | 0.000 | λ_(burrows + avasmam + soilhum + diswater)_ | -195.024 | -1.275 | 0.700 | 0.000 |
|  | λ_(shrcover + cancover)_ | -77.317 | -3.724 | 2.466 | 0.000 | λ_(shrcover + cancover + burrows + avasmam + soilhum + diswater)_ | -196.494 | -2.745 | 1.284 | 0.000 |
|  | λ_(avasmam + soilhum)_ | -78.609 | -5.016 | 2.811 | 0.000 | λ_(shrcover + cancover)_ | -212.836 | -19.087 | 6.134 | 0.000 |
|  | λ_(burrows + avasmam + soilhum + diswater)_ | -79.069 | -5.476 | 2.189 | 0.000 | λ_(shrcover + cancover + burrows)_ | -214.223 | -20.473 | 6.318 | 0.000 |
|  | *Combination* | | | | |  |  |  |  |  |
|  | λ_(cancover + avasmam + soilhum + ocelots)_ | -72.760 | 0.000 | 0.000 | 1.000 | λ_(avasmam + diswater + coyotes)_ | -193.177 | 0.000 | 0.000 | 0.823 |
|  | λ_(shrcover + cancover + competitor)_ | -78.131 | -5.371 | 2.110 | 0.000 | λ_(avasmam + coyotes)_ | -193.901 | -0.723 | 1.363 | 0.001 |
|  | λ_(shrcover + cancover + burrows + avasmam + competitor)_ | -78.639 | -5.879 | 2.797 | 0.000 | λ_(avasmam + coyotes + ocelots)_ | -194.187 | -1.010 | 1.737 | 0.154 |
|  | λ_(shrcover + cancover + coyotes + ocelots)_ | -79.198 | -6.438 | 2.501 | 0.000 | λ_(avasmam + burrows + competitor)_ | -196.495 | -3.317 | 2.534 | 0.022 |
|  | λ_(avasmam + burrows + competitor)_ | -80.428 | -7.668 | 3.050 | 0.000 | λ_(shrcover + cancover + burrows + avasmam + competitor)_ | -197.942 | -4.765 | 2.883 | 0.000 |
|  | λ_(avasmam + coyotes + ocelots)_ | -82.655 | -9.895 | 2.876 | 0.000 | λ_(shrcover + cancover + coyotes + ocelots)_ | -212.512 | -19.335 | 6.535 | 0.000 |
|  | λ_(diswater + coyotes + ocelots)_ | -82.755 | -9.995 | 2.807 | 0.000 | λ_(shrcover + cancover + competitor)_ | -213.796 | -20.619 | 6.462 | 0.000 |
| **Dry season 2020** | *Interespecific interactions* | | | | |  |  |  |  |  |
|  | λ_(coyotes)_ | -30.002 | 0.000 | 0.000 | 0.716 | λ_(coyotes)_ | -77.968 | 0.000 | 0.000 | 1.000 |
|  | λ_(competitor)_ | -30.947 | -0.945 | 0.729 | 0.000 | λ_(coyotes + ocelots)_ | -79.333 | -1.365 | 0.626 | 0.000 |
|  | λ_(ocelots)_ | -31.182 | -1.180 | 2.175 | 0.000 | λ_(competitor)_ | -80.261 | -2.292 | 2.012 | 0.000 |
|  | λ_(dogs)_ | -31.256 | -1.254 | 2.606 | 0.284 | λ_(competitor + coyotes + ocelots + dogs)_ | -80.547 | -2.579 | 1.651 | 0.000 |
|  | λ_(coyotes + ocelots)_ | -31.619 | -1.617 | 1.251 | 0.000 | λ_(ocelots)_ | -80.565 | -2.597 | 1.691 | 0.000 |
|  | λ_(coyotes + ocelots + dogs)_ | -32.989 | -2.987 | 2.478 | 0.000 | λ_(dogs)_ | -80.641 | -2.673 | 1.552 | 0.000 |
|  | λ_(competitor + coyotes + ocelots + dogs)_ | -34.322 | -4.320 | 2.755 | 0.000 | λ_(coyotes + ocelots + dogs)_ | -80.858 | -2.890 | 0.828 | 0.000 |
|  | *Resource availability and habitat complexity* | | | | |  |  |  |  |  |
|  | λ_(shrcover)_ | -30.229 | 0.000 | 0.000 | 1.000 | λ_(shrcover + avasmam)_ | -72.616 | 0.000 | 0.000 | 1.000 |
|  | λ_(shrcover + cancover)_ | -31.173 | -0.943 | 0.390 | 0.000 | λ_(shrcover + cancover + burrows)_ | -75.110 | -2.494 | 1.769 | 0.000 |
|  | λ_(shrcover + cancover + burrows)_ | -32.230 | -2.000 | 0.631 | 0.000 | λ_(shrcover + cancover + burrows + avasmam + soilhum + diswater)_ | -75.623 | -3.007 | 1.787 | 0.000 |
|  | λ_(avasmam + soilhum)_ | -32.763 | -2.534 | 1.223 | 0.000 | λ_(shrcover + cancover)_ | -75.965 | -3.349 | 2.028 | 0.000 |
|  | λ_(burrows + avasmam + soilhum + diswater)_ | -34.035 | -3.805 | 2.008 | 0.000 | λ_(avasmam + diswater)_ | -76.562 | -3.946 | 2.099 | 0.000 |
|  | λ_(shrcover + cancover + burrows + avasmam + soilhum + diswater)_ | -35.521 | -5.292 | 3.444 | 0.000 | λ_(burrows + avasmam + soilhum + diswater)_ | -77.914 | -5.298 | 2.147 | 0.000 |
|  | *Combination* | | | | |  |  |  |  |  |
|  | λ_(shrcover + coyotes)_ | -29.581 | 0.000 | 0.000 | 1.000 | λ_(shrcover + avasmam + coyotes)_ | -73.298 | 0.000 | 0.000 | 1.000 |
|  | λ_(shrcover + cancover + competitor + coyotes)_ | -31.248 | -1.667 | 1.201 | 0.000 | λ_(shrcover + cancover + coyotes + ocelots)_ | -75.397 | -2.099 | 1.490 | 0.000 |
|  | λ_(shrcover + cancover + competitor)_ | -31.302 | -1.722 | 1.055 | 0.000 | λ_(shrcover + cancover + avasmam + coyotes + ocelots)_ | -75.594 | -2.296 | 0.605 | 0.000 |
|  | λ_(shrcover + cancover + coyotes + ocelots)_ | -31.776 | -2.195 | 1.197 | 0.000 | λ_(avasmam + diswater + competitor)_ | -76.929 | -3.631 | 2.633 | 0.000 |
|  | λ_(avasmam + coyotes + ocelots)_ | -32.716 | -3.135 | 1.894 | 0.000 | λ_(shrcover + cancover + burrows + competitor)_ | -76.954 | -3.656 | 1.942 | 0.000 |
|  | λ_(burrows + avasmam + competitor)_ | -33.452 | -3.872 | 1.506 | 0.000 | λ_(shrcover + cancover + competitor)_ | -77.041 | -3.743 | 2.185 | 0.000 |
|  |  |  |  |  |  | λ_(avasmam + coyotes + ocelots)_ | -77.906 | -4.608 | 2.044 | 0.000 |

elpd, expected log pointwise predictive density; Δelpd, pairwise differences in elpd (relative to the top model); SE[Δelpd], standard error of Δelpd; ω, model weight.

^a^ The key to covariate abbreviations is: competitor, presence of competitors; coyotes, presence of coyotes; dogs, presence of dogs; ocelots, presence of ocelots; burrows, potential burrows; avasmam, availability of small mammals; soilhum, soil humidity; dishwater, distance to the nearest water source; shrcover, shrub cover; cancover, canopy cover.
