# Supplementary material for: Availability of alternative prey rather than intraguild interactions determines the local abundance of two understudied and threatened small carnivore species
Source: PLoS One. 2024 Nov 8;19(11):e0310021. doi: 10.1371/journal.pone.0310021 (PMC11548751; doi:10.1371/journal.pone.0310021)
Supplement: S6 Table — (DOCX) [file pone.0310021.s007.docx]

**S6 Table. Parameter estimates of spatial models incorporating the spatial random effect to determine the average abundance per site of hog-nosed skunks at the each surveyed season.**

|  | **Covariate^a^** | **β** | **SD** | **2.5%** | **97.5%** | **n_eff** | **Rhat** |
| --- | --- | --- | --- | --- | --- | --- | --- |
| **Dry season 2019** | *Abundance* |  |  |  |  |  |  |
|  | Intercept | -0.512 | 0.952 | -2.381 | 1.35 | 1005 | 1 |
|  | diswater | 1.223 | 0.581 | 0.268 | 2.51 | 1184 | 1 |
|  | RSR [tau]^b^ | 99.839 | 136.852 | 0.216 | 492.89 | 279 | 1 |
|  | *Detection* |  |  |  |  |  |  |
|  | Intercept | -3.756 | 0.831 | -5.403 | -2.273 | 919 | 1 |
| **Rainy season 2019** | *Abundance* |  |  |  |  |  |  |
|  | Intercept | -1.414 | 0.826 | -3.172 | 0.105 | 1688 | 1 |
|  | avamam | 0.869 | 0.291 | 0.299 | 1.451 | 1741 | 1 |
|  | soilhum | -1.287 | 0.589 | -2.557 | -0.233 | 1865 | 1 |
|  | cancover | -1.029 | 0.322 | -1.701 | -0.435 | 1545 | 1 |
|  | ocelots | 0.308 | 0.201 | -0.148 | 0.651 | 2102 | 1 |
|  | RSR [tau] | 101.678 | 140.752 | 0.633 | 504.897 | 693 | 1.01 |
|  | *Detection* |  |  |  |  |  |  |
|  | Intercept | -3.677 | 0.647 | -4.856 | -2.374 | 2538 | 1 |
|  | effort | 1.32 | 0.728 | -0.016 | 2.846 | 2334 | 1 |
| **Dry season 2020** | *Abundance* |  |  |  |  |  |  |
|  | Intercept | -0.785 | 0.985 | -2.693 | 1.246 | 1272 | 1 |
|  | coyotes | -1.151 | 0.75 | -2.973 | 0.026 | 1815 | 1 |
|  | shrcover | -1.43 | 1.034 | -3.646 | 0.277 | 1691 | 1 |
|  | RSR [tau] | 96.283 | 129.998 | 0.359 | 451.353 | 806 | 1 |
|  | *Detection* |  |  |  |  |  |  |
|  | Intercept | -2.997 | 0.876 | -4.785 | -1.487 | 1595 | 1 |

SD, Standard Deviation; n_eff, effective sample size; Rhat, diagnostic statistic (< 1.1); RSR, Restricted Spatial Regression.

^a^ The key to covariate abbreviations is: coyotes, presence of coyotes; ocelots, presence of ocelots; avasmam, availability of small mammals; soilhum, soil humidity; diswater, distance to the nearest water source; shrcover, shrub cover; cancover, canopy cover; effort, sampling effort; lunillu, lunar illumination.

^b^ Higher values of tau indicate a lower spatial relationship between the n sites, that is, a lower statistical significance of a spatial random effect [1, 2]. 1. Paddock SM, Leininger TJ, Hunter SB. Bayesian restricted spatial regression for examining session features and patient outcomes in open-enrollment group therapy studies. Stat Med. 2016; 35(1):97–114. 2. Kellner K. Spatial Models in ubms. 2024. https://cran.r-project.org/web/packages/ubms/vignettes/spatial-models.html
