# Supplementary material for: Availability of alternative prey rather than intraguild interactions determines the local abundance of two understudied and threatened small carnivore species
Source: PLoS One. 2024 Nov 8;19(11):e0310021. doi: 10.1371/journal.pone.0310021 (PMC11548751; doi:10.1371/journal.pone.0310021)
Supplement: S7 Table — (DOCX) [file pone.0310021.s008.docx]

**S7 Table. Parameter estimates of top-ranked Royle-Nichols models explaining the abundance (λ) of skunk species at the each surveyed season.**

|  | **American hog-nosed skunk** | | | | | | | **Pygmy spotted skunk** | | | | | | |
| --- | --- | --- | --- | --- | --- | --- | --- | --- | --- | --- | --- | --- | --- | --- |
|  | **Covariate^a^** | **β** | **SD** | **2.5%** | **97.5%** | **n_eff** | **Rhat** | **Covariate^a^** | **β** | **SD** | **2.5 %** | **97.5 %** | **n_eff** | **Rhat** |
| **Dry season 2019** | *Abundance* |  |  |  |  |  |  | *Abundance* |  |  |  |  |  |  |
|  | Intercept | -0.474 | 0.913 | -2.290 | 1.290 | 769 | 1 | Intercept | -0.251 | 0.335 | -0.919 | 0.407 | 1310 | 1 |
|  | diswater | 1.223 | 0.549 | 0.280 | 2.420 | 799 | 1 | avamam | 0.633 | 0.135 | 0.356 | 0.895 | 1837 | 1 |
|  |  |  |  |  |  |  |  | diswater | 0.751 | 0.255 | 0.274 | 1.290 | 1575 | 1 |
|  | *Detection* |  |  |  |  |  |  | *Detection* |  |  |  |  |  |  |
|  | Intercept | -3.810 | 0.811 | -5.340 | -2.270 | 919 | 1 | Intercept | -1.400 | 0.316 | -2.060 | -0.832 | 1491 | 1 |
| **Rainy season 2019** | *Abundance* |  |  |  |  |  |  | *Abundance* |  |  |  |  |  |  |
|  | Intercept | -1.427 | 0.872 | -3.285 | 0.183 | 898 | 1 | Intercept | 0.673 | 0.250 | 0.177 | 1.160 | 1580 | 1 |
|  | avamam | 0.876 | 0.300 | 0.293 | 1.490 | 1019 | 1 | avamam | 0.653 | 0.090 | 0.477 | 0.829 | 1904 | 1 |
|  | soilhum | -1.295 | 0.611 | -2.638 | -0.252 | 1102 | 0.999 | diswater | -0.239 | 0.139 | -0.513 | 0.040 | 2571 | 1 |
|  | cancover | -1.039 | 0.333 | -1.742 | -0.433 | 949 | 1 | coyotes | -0.332 | 0.187 | -0.720 | 0.001 | 2453 | 1 |
|  | ocelots | 0.311 | 0.198 | -0.127 | 0.658 | 1006 | 1 |  |  |  |  |  |  |  |
|  | *Detection* |  |  |  |  |  |  | *Detection* |  |  |  |  |  |  |
|  | Intercept | -3.680 | 0.640 | -4.914 | -2.380 | 1099 | 1 | Intercept | -1.920 | 0.249 | -2.410 | -1.432 | 1783 | 1 |
|  | effort | 1.320 | 0.730 | -0.065 | 2.810 | 1051 | 1 | lunillu | -0.230 | 0.115 | -0.460 | -0.002 | 2607 | 1 |
| **Dry season 2020** | *Abundance* |  |  |  |  |  |  | *Abundance* |  |  |  |  |  |  |
|  | Intercept | -0.722 | 0.976 | -2.560 | 1.232 | 1144 | 1 | Intercept | -0.226 | 0.501 | -1.166 | 0.812 | 1133 | 1 |
|  | coyotes | -1.114 | 0.711 | -2.710 | 0.041 | 1382 | 1 | avamam | 0.562 | 0.220 | 0.097 | 0.987 | 1615 | 1 |
|  | shrcover | -1.360 | 1.011 | -3.550 | 0.288 | 1481 | 1 | shrcover | -1.646 | 0.602 | -2.862 | -0.499 | 1643 | 1 |
|  | *Detection* |  |  |  |  |  |  | *Detection* |  |  |  |  |  |  |
|  | Intercept | -3.010 | 0.870 | -4.820 | -1.560 | 1250 | 1 | Intercept | -1.200 | 0.527 | -2.270 | -0.257 | 1180 | 1 |
|  |  |  |  |  |  |  |  | effort | -0.689 | 0.304 | -1.300 | -0.120 | 1392 | 1 |

SD, Standard Deviation; n_eff, effective sample size; Rhat, diagnostic statistic (< 1.1).

^a^ The key to covariate abbreviations is: coyotes, presence of coyotes; ocelots, presence of ocelots; avasmam, availability of small mammals; soilhum, soil humidity; diswater, distance to the nearest water source; shrcover, shrub cover; cancover, canopy cover; effort, sampling effort; lunillu, lunar illumination.
