# Supplementary material for: Availability of alternative prey rather than intraguild interactions determines the local abundance of two understudied and threatened small carnivore species
Source: PLoS One. 2024 Nov 8;19(11):e0310021. doi: 10.1371/journal.pone.0310021 (PMC11548751; doi:10.1371/journal.pone.0310021)
Supplement: Attachment — Submitted filename: Response to Reviewers.docx [file pone.0310021.s010.docx]

| **Date** | August 7, 2024 |
| --- | --- |
| **Manuscript ID:** | PONE-D-23-33252R1 |
| **Manuscript Title** | Availability of alternative prey rather than intraguild interactions determines the local abundance of two understudied and threatened small carnivore species |
| **Authors** | Alejandro Hernández-Sánchez, Antonio Santos-Moreno |

| **Observation/Comment** | **Response/Reply** |
| --- | --- |
| **Methods** |  |
| Lines 313-314: Did you only use the negative binomial, or were there some that you used the Poisson for? Or did you decide based on AIC/other criteria? It’s not quite clear to me. Only NB is mentioned in the following paragraph, but please mention the Poisson if you fit some models with that distribution, or how you chose between the two for each model. | We clarified this point and added information for a better understanding of Poisson and Negative Binomial model fitting and selection. (Line: 321-324; 335-338). Also, see the S2 Table of the supplementary files. |
| Line 317-319: Close on the explanation for NB instead of Poisson – note that the Poisson can’t explain extra variability, so that’s why we consider other distributions for a lot of highly variable ecological data. Consider this wording instead: “The Negative Binomial distribution includes a dispersion parameter that allows it to explain more variability than the Poisson distribution” | We agree with you. Done without problem.  (Line: 317-318) |
| **Results** |  |
| Minor comment, but consider having BCI’s as -0.5 to -0.2 instead of -0.5 - -0.2. The style of the dashes makes it hard to pick out the negatives in the second number. Alternatively or in addition, you could add a + before positive numbers. But also ok if you choose to leave this as-is. | We followed your recommendation throughout the Results section. We changed the dashes to "to" in all BCI values to homogenize the text. |
| **Figures** |  |
| We corrected our figures with the help of the digital diagnostic tool Preflight Analysis and Conversion Engine (PACE), so we uploaded the files again. | |
